# Analyzing Severe Acute Kidney Injury Using the Renal Angina Index

**DOI:** 10.7759/cureus.110835

**Published:** 2026-06-14

**Authors:** Rakasi S Reddy, Mamata Devi Mohanty, Sagar Parida, Sree Vaishnavi C Peddi, Basudev Biswal

**Affiliations:** 1 Paediatrics, Institute of Medical Sciences and SUM Hospital, Siksha 'O' Anusandhan Deemed to be University, Bhubaneswar, IND; 2 Pediatric Critical Care, Institute of Medical Sciences and SUM Hospital, Siksha 'O' Anusandhan Deemed to be University, Bhubaneswar, IND; 3 Paediatrics and Child Health, Institute of Medical Sciences and SUM Hospital, Siksha 'O' Anusandhan Deemed to be University, Bhubaneswar, IND; 4 Pediatrics, Institute of Medical Sciences and SUM Hospital, Siksha 'O' Anusandhan Deemed to be University, Bhubaneswar, IND

**Keywords:** acute kidney injury, critical care, pediatric, renal angina index, risk predictor

## Abstract

Introduction: Acute kidney injury (AKI) is one of the most common complications in critically ill children admitted to the pediatric intensive care unit (PICU). The Renal Angina Index (RAI) is one of the newer effective tools for predicting AKI. It is assessed at the time of admission to the PICU and is used to determine the risk of subsequent severe AKI. The RAI score may be calculated by combining objective signs of kidney dysfunction and patient risk factors.

Objective: This study aims to evaluate the early prediction of severe AKI in patients using the RAI.

Methods: This was a prospective observational study conducted in the PICU of the Institute of Medical Sciences and SUM Hospital from 2020 to 2022. Children aged 1 month to 14 years were included. RAI scoring was performed on day 0, and the development of AKI was assessed on day 3.

Results: The mean (±SD) age of the study patients was 5.25 (±4.03) years. RAI positivity was seen in 44.9% of patients, of whom AKI developed in 37.7%. A larger proportion of RAI-positive patients required mechanical ventilation (71%) compared with RAI-negative patients (9.6%). The mean (±SD) duration of PICU stay in the RAI-positive group was 10.38 (±10.24) days compared with 6.95 (±4.73) days in the RAI-negative group. The majority of RAI-positive patients developed AKI (32.3% stage 1 AKI, 24.7% stage 2 AKI, and 16.1% stage 3 AKI), which was significant compared with RAI-negative patients (7.9% stage 1 AKI, 0.9% stage 2 AKI, and 0% stage 3 AKI). Mortality was observed in 20.4% of RAI-positive patients versus 7% of RAI-negative patients.

Conclusions: The RAI score is a reliable tool for predicting AKI in critically ill children.

## Introduction

Acute kidney injury (AKI) is characterized by a rapid loss of renal function, and diagnosis is based on an increase in serum creatinine, a decrease in urine output, or both [1]. AKI is common in critically ill pediatric patients and is associated with increased mortality, prolonged hospitalization, increased comorbidities, and the development of chronic kidney disease [2-4]. Recent research has focused on the early identification of AKI using different serum and/or urine biomarkers (neutrophil gelatinase-associated lipocalin, interleukin-18, kidney injury molecule-1, liver fatty acid-binding protein, etc.) [5-8]. The concept of Renal Angina Index (RAI) scoring was introduced to strengthen the clinical applicability of serum biomarkers and has recently been validated for the early detection of severe AKI [8-10].

RAI is a combination score of AKI risk strata and clinical injury signs of AKI. For AKI risk strata, moderate risk includes pediatric intensive care unit (PICU) admission (score 1), high risk includes a history of bone marrow or solid organ transplantation (score 3), and very high risk includes ventilation and inotrope use (score 5). For clinical injury signs of AKI, <5% fluid overload or no change in estimated creatinine clearance (eCrCl) is assigned a score of 1; 5-10% fluid overload or a decrease in eCrCl by 0-24% is assigned a score of 2; 10-15% fluid overload or a decrease in eCrCl by 25-49% is assigned a score of 4; and ≥15% fluid overload or a decrease in eCrCl by ≥50% is assigned a score of 8.

This score has been validated in multiple studies of different populations [10-12]. In our study, RAI scoring was performed on day 0 of hospitalization in children admitted to the PICU, and a score >8 was considered positive for predicting the development of severe AKI (KDIGO (Kidney Disease: Improving Global Outcomes) classification) on day 3 [11-12]. Recent research has focused on the early detection of AKI to enable timely intervention and prevent severe AKI.

## Materials and methods

Our study was a prospective observational study conducted in the PICU of the Institute of Medical Sciences and SUM Hospital from 2020 to 2022. Ethical clearance was obtained from the Institutional Ethics Committee. All children of either sex admitted to the PICU between the ages of 1 month and 14 years were included in the study. Consecutive sampling was performed at the time of enrollment, and written informed consent was obtained from the parents. Children with previous renal diseases, those admitted to the PICU for less than 72 hours, and those diagnosed with AKI stage 2 or above at the time of admission were excluded. Children who were discharged or died within 72 hours of admission were also excluded from the study. The first day of PICU admission and 72-96 hours after PICU admission were considered day 0 and day 3, respectively. Detailed clinical symptoms at presentation, anthropometry, vital signs, 24-hour urine output, clinical examination findings, and laboratory investigations were documented. All children were evaluated and managed as per hospital protocol. RAI scoring was performed on day 0, and those children who developed AKI were classified according to the KDIGO classification on day 3.

RAI was calculated based on the risk and injury criteria. Risk factors were assigned scores of 1, 3, or 5, with 1 being the lowest and 5 being the highest. Risk criteria were categorized into three scores. Admission to the PICU was assigned a score of 1, stem cell transplantation or solid organ transplantation was assigned a score of 3, and inotropic support or mechanical ventilatory support was assigned a score of 5. The score was calculated at the time of admission or within 12 hours thereafter. Injury criteria were based on baseline serum creatinine or fluid overload status within 12 hours after admission. Creatinine clearance was estimated using the Schwartz formula, and a score was assigned based on the decrease in creatinine clearance. No change was assigned a score of 0, a 0-24.9% decrease was assigned a score of 2, a 25-50% decrease was assigned a score of 4, and a >50% decrease was assigned a score of 8. The lowest serum creatinine level within the 3 months before admission was considered the baseline serum creatinine level. Fluid overload (FO%) was estimated as follows.

$\text{Fluid overload} = \frac{\text{Fluid intake (mL)} &minus; \text{Fluid output (mL)}}{\text{Patient weight (g)}}\times 100$

RAI was calculated as the product of the risk score and injury score and was defined as positive if the score was ≥8 on day 0. The proportion of children developing AKI on day 3 was analyzed according to day 0 RAI-positive and RAI-negative scores.

Statistical analysis

Categorical variables were presented as frequencies and proportions, and continuous variables were presented as mean (± standard deviation). The chi-square test was used to assess statistical significance. A p-value <0.05 was considered statistically significant. IBM SPSS Statistics for Windows, Version 21 (Released 2012; IBM Corp., Armonk, New York) was used for statistical analysis.

## Results

Out of 290 patients, 207 were included in the study and 83 were excluded (32 had severe AKI at admission, 26 did not provide consent, 12 had a duration of stay <72 hours, and 13 had CKD). The mean (±SD) age of the study patients was 5.25 (±4.03) years. Among the study population, the majority (44.9%) were in the 1-5-year subgroup, followed by the >5-year subgroup (40.1%) and the <1-year subgroup (15%). Out of 207 patients, 44.9% were RAI positive (RAI score >8) and 55.1% were RAI negative (RAI score ≤8) on day 0. Table 1 shows the baseline characteristics of the RAI-positive and RAI-negative groups.

**Table 1:** Comparison of parameters among RAI-positive and RAI-negative children. RAI: Renal Angina Index, AKI: acute kidney injury.

Parameters	RAI-positive (N = 93)	RAI-negative (N = 114)	P-value
Male	59 (45%)	72 (55%)	
Female	34 (44.7%)	42 (55.3%)	
No AKI	25(26.9%)	104 (91.2%)	
Stage 1 AKI	30 (32.3%)	9 (7.9%)	
Stage 2 AKI	23 (24.7%)	1 (0.9%)	<0.001
Stage 3 AKI	15 (16.1%)	0	
Inotropes	61 (65.6%)	25 (21.9%)	
Multiple inotropes	50 (53.8%)	16 (14%)	
Single inotropes	11 (11.8%)	9 (7.9%)	<0.001
Mechanical ventilation	66 (71%)	11 (9.6%)	<0.001
Nephrotoxic drugs	18 (62.1%)	11 (37.9%)	<0.005
Mortality	19 (20.4%)	8 (7.0%)	0.004

In this study, 62.3% of patients had no AKI, 18.8% had stage 1 AKI, 11.6% had stage 2 AKI, and 7.2% had stage 3 AKI. A larger proportion of RAI-positive patients required mechanical ventilation (71%) compared with RAI-negative patients (9.6%). The mean (±SD) duration of PICU stay in the study population was 8.49 (±7.87) days. The mean (±SD) duration of PICU stay observed in the RAI-positive group was 10.38 (±10.24) days compared with 6.95 (±4.73) days in the RAI-negative group. A higher percentage (53.8%) of children with RAI-positive scores required multiple inotropes. Use of nephrotoxic drugs was more common in the RAI-positive group (62.1%) than in the RAI-negative group (42.1%). The majority of RAI-positive patients developed AKI (32.3% stage 1 AKI, 24.7% stage 2 AKI, and 16.1% stage 3 AKI), which was significant compared with RAI-negative patients (7.9% stage 1 AKI, 0.9% stage 2 AKI, and 0% stage 3 AKI). Mortality was 20.4% in RAI-positive patients and 7% in RAI-negative patients. Sepsis with multiorgan dysfunction was the most common cause of mortality in our study.

In our study, the RAI scores were divided into five subcategories: 1-8, 9-16, 17-24, 25-32, and 33-40 (Table 2). The highest proportion of patients with RAI scores of 1-8 did not develop AKI (88.1%). RAI scores >24 were associated with a higher proportion of stage 2 AKI (66.7%) and stage 3 AKI (33.3%). Central nervous system (31.9%) and respiratory system (20.3%) pathologies were the predominant organ systems involved in the study population. Other systemic causes involved the rheumatic, cardiovascular, gastrointestinal, and hematological systems. 

**Table 2:** Comparison of RAI scores on day 0 with severity of AKI on Day 3. RAI: Renal Angina Index, AKI: acute kidney injury.

RAI score	AKI				P-value
	No AKI	Stage 1	Stage 2	Stage 3	
1 to 8 (n = 118)	104 (88.1%)	9 (7.6%)	3 (2.5%)	2 (1.7%)	<0.001
9 to 16 (n = 42)	24 (57.1%)	11 (26.2%)	3 (7.1%)	4 (9.5%)	
17 to 24 (n = 32)	1 (3.1%)	19 (59.4%)	8 (25%)	4 (12.5%)	
>24 (n = 15)	0 (0%)	0 (0%)	10 (66.7%)	5 (33.3%)	

## Discussion

This prospective study demonstrated that a positive RAI on day 0 of admission to the critical care unit is significantly associated with the development of AKI on day 3. This study is comparable to other studies in terms of similar populations [13,14]. Since this study integrates patient risk factors with early signs of AKI, RAI can easily predict severe AKI in high-risk patients and can effectively rule it out in low-risk patients. Thus, early preventive intervention and timely nephrology referral can be undertaken. This study strengthens previous research on the clinical utility of RAI in predicting AKI. Though our study had many limitations, it broadens knowledge of RAI in diverse subgroups of the Indian population.

The mean (±SD) age of the study population was 5.25 (±4.03) years, among whom 15% were <1 year, 44.9% were 1-5 years, and 40.1% were >5 years. Similar to our study, Kaur et al., in a prospective observational study, reported a mean age of 5.8 years [15]. In another study, Gawadia et al. reported positive RAI results in 59% and 34% of males and females, respectively [13]. Another similar study comparing RAI-positive and RAI-negative patients reported proportions of 63% and 71% males, respectively [16]. Our study also demonstrated a slight male predominance. Although it is unclear whether this is a result of disease epidemiology or earlier hospitalization of male children due to local taboos, particularly in the Indian community, similar observations have been reported [13].

RAI positivity in this study was 44.9%, which is similar to that reported in a previous study [13]. However, two other studies by Basu et al. and Matsuura et al. reported only 17% positivity [17,18]. In the present study, we divided the proportion of AKI among RAI-positive and RAI-negative patients. Among RAI-positive patients, 26.9% did not have AKI, 32.3% had stage 1 AKI, 24.7% had stage 2 AKI, and 16.1% had stage 3 AKI. Among RAI-negative patients, 91.2% did not have AKI, 7.9% had stage 1 AKI, 0.9% had stage 2 AKI, and none had stage 3 AKI; this difference was statistically significant (p<0.05). Gawadia et al. reported similar significant findings [13]. In the current study, we evaluated the applicability of RAI in a tertiary care hospital in a developing country and showed that it can be a useful predictor of AKI.

In our study, 31.9% required multiple inotropes and 9.7% required single inotropic support. Kaur et al. reported 26.1%, and Punam Bajracharya et al. reported approximately 17% requiring inotropic support [15,19]. Norepinephrine was the most commonly used inotrope in our PICU. Among RAI-positive patients, 65.5% required inotropic support, compared with 21.9% of RAI-negative patients. Similarly, Gawadia et al. reported rates of 78% and 32%, respectively [13]. Huang et al., in a retrospective study, reported that RAI-positive patients required more inotropic support [16]. This may be due to a higher prevalence of sepsis or MODS.

Among RAI-positive patients, the proportion of children who required ventilatory support was 71.5%, whereas among RAI-negative patients, 9.6% required ventilatory support. Gawadia et al. and Huang et al. also reported findings similar to ours [13,16]. Among RAI-positive patients, 20.4% died. Among patients with RAI scores between 1 and 8, 88.1% did not develop AKI. Among patients with RAI scores between 9 and 16, 57.1% did not develop AKI and 26.2% had stage 1 AKI. The highest proportion of severe AKI (stage 3) occurred in patients with RAI scores of 17-23 and >24. Hence, there was a statistically significant difference in the proportion of AKI according to RAI score (p<0.05). This study strengthens findings from previous research suggesting that higher RAI scores are associated with a greater proportion of AKI [13,14].

Our study further strengthens previous research regarding the clinical utility of RAI in predicting AKI in children. It also supports the association between higher RAI scores and the use of multiple inotropes, an increased requirement for mechanical ventilation, prolonged PICU stay, and an increased risk of developing severe AKI. However, our study had some limitations, including a small sample size, single-center bias, and a lack of biomarker comparison. Multicenter research with a larger sample of diverse, heterogeneous groups of children is needed to generalize the results. Our study did not compare day 0, day 3, and day 7 RAI scores and their correlation with AKI. It also does not provide conclusive strategies to reduce the risk of AKI. In addition, we did not compare PICU mortality or morbidity scores, which might have helped delineate comorbidities as confounding factors associated with higher RAI scores and the subsequent development of severe AKI.

## Conclusions

The RAI score predicts severe AKI in high-risk patients and demonstrates a high negative predictive value for the development of severe AKI in low-risk patients. It can serve as a reliable bedside tool for predicting AKI, thereby overcoming the limitations of serum creatinine in the early stages. It provides an early opportunity to identify patients at risk for severe AKI, enabling healthcare providers to implement focused preventive interventions for high-risk critically ill patients.
